# Draft Genome Sequence of a Mexican Community-Associated Methicillin-Resistant *Staphylococcus epidermidis* Strain

**DOI:** 10.1128/genomeA.01236-17

**Published:** 2017-11-02

**Authors:** José Antonio Magaña-Lizárraga, Jessica Victoria Hernández-Peinado, Yesmi Patricia Ahumada-Santos, Jesús Ricardo Parra-Unda, Magdalena de J. Uribe-Beltrán, Bruno Gómez-Gil, María Elena Báez-Flores

**Affiliations:** a Unidad de Investigaciones en Salud Pública Dra. Kaethe Willms, Facultad de Ciencias Químico Biológicas, Universidad Autónoma de Sinaloa, Culiacán, Sinaloa, Mexico; b Centro de Investigación en Alimentación y Desarrollo, A. C. Mazatlán, Sinaloa, Mexico

## Abstract

We report here the first draft genome sequence of a Mexican communitarian methicillin-resistant *Staphylococcus epidermidis* (MRSE) strain whose genome harbors a wide variety of resistance determinants. The availability of this genome will allow the study of antibiotic resistance in Mexican staphylococci from a genomic perspective.

## GENOME ANNOUNCEMENT

The overuse of methicillin and other penicillins has led to the emergence of methicillin-resistant *Staphylococcus epidermidis* (MRSE) (1). This formerly innocuous commensal on human skin and nasopharynx microbiota (2) is now considered an opportunistic nosocomial pathogen as relevant as *Staphylococcus aureus* (3), particularly in neonates and patients who are immunocompromised or have implanted devices (4). *S. epidermidis* is a major cause of hospital-acquired infections (5) in that it is associated with high rates of morbimortality worldwide. The genomic study of the multiresistance phenotype of a Mexican strain of *S. epidermidis* is greatly needed.

Here, we present the genome sequence of *S. epidermidis* MRSE 52-2, an isolate recovered from a pharyngeal exudate from a healthy child in a day care center. The isolate showed resistance to macrolides, lincosamides, and streptogramins (Vitek 2 Systems: 06.01). The MRSE phenotype was settled by *mecA* gene amplification (6).

The genome sequencing of *S. epidermidis* was performed using the Ion Torrent PGM (Life Technologies, Carlsbad, CA), with 200-bp chemistry on a 318 Chip kit. The raw sequence data contained 1,540,332 reads, with a mean length of 203 bp and a total output of 312.9 Mb. The reads were trimmed with PrinSeq (7) and assembled with Newbler version 2.3, resulting in 146 contigs with a maximum length of 188,073 bp, an *N*_50_ of 66,734 bp, and a predicted genome size of 2,564,417 bp. The G+C content was 31.86%. Fifty percent of the genome is contained in 11 contigs.

Open reading frames (ORFs), rRNA, and tRNA genes were annotated by the NCBI Prokaryotic Genome Annotation Pipeline (PGAP; version 3.3) with GeneMarkS+ and the best-placed reference-protein set method (8). Additionally, reads were annotated by Rapid Annotation using Subsystem Technology (RAST) server (9). The PGAP analysis revealed 2,570 genes, 2,509 coding sequences (CDS), 2,296 coding CDSs, 61 RNA genes (4 rRNAs, 53 tRNAs, and 4 noncoding RNAs [ncRNAs]), and 213 pseudogenes. According the RAST nearest-neighbor analysis, the closest genome is *Staphylococcus epidermidis* RP62A. Also, the RAST analysis found 383 subsystem features, of which 40 corresponded with resistance to antibiotics (teicoplanin, fluoroquinolones, fosfomycin, and beta-lactams) and toxic substances (copper, cobalt, zinc, cadmium, and arsenic).

By a ResFinder (10) search, four resistance genes were identified [*mecA*, *blaZ*, *fosA*, and *msr*(A)] in the MRSE 52-2 genome.

### Accession number(s).

This whole-genome shotgun project has been deposited at DDBJ/ENA/GenBank under the accession number NTLC00000000. The version described in this paper is the first version, NTLC01000000.
